# Supplementary material for: Environmental change makes robust ecological networks fragile
Source: Nat Commun. 2016 Aug 11;7:12462. doi: 10.1038/ncomms12462 (PMC4987532; doi:10.1038/ncomms12462)
Supplement: Supplementary Information — Supplementary Figures 1-9, Supplementary Tables 1-2 [file ncomms12462-s1.pdf]

Supplementary information

Supplementary Figures

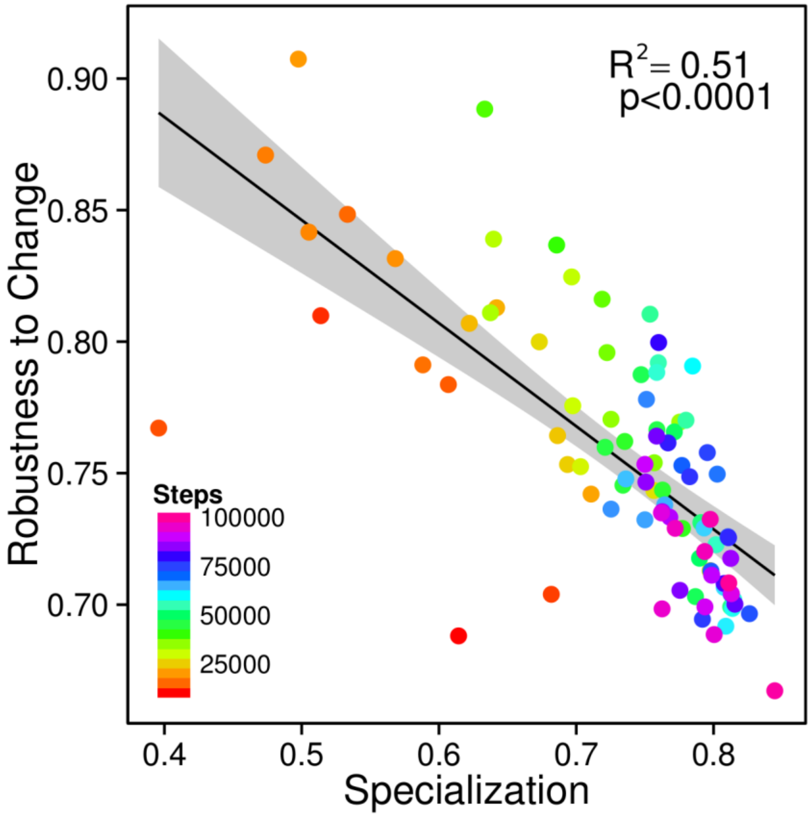

Supplementary Figure 1 | Specialization in digital host-parasite assemblages increases over time, while robustness to novel conditions decreases. Specialization was computed, in each digital host-parasite assemblage, as 1 minus the fraction of hosts used by all parasites.

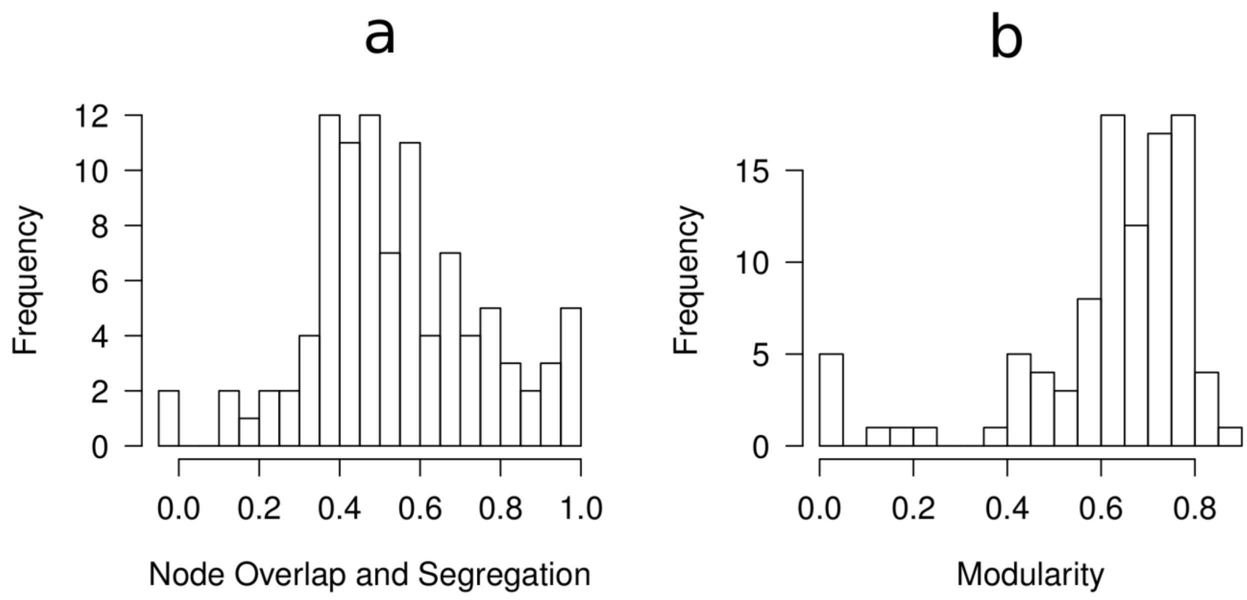

Supplementary Figure 2 | Distributions of node overlap and segregation (nestedness) values and modularity in the 100 co-evolved host-parasite networks. (a) Most networks tends toward randomness (0) or nestedness (1), although no network tends toward segregation (< 0). (**b**) Most networks show high modularity.

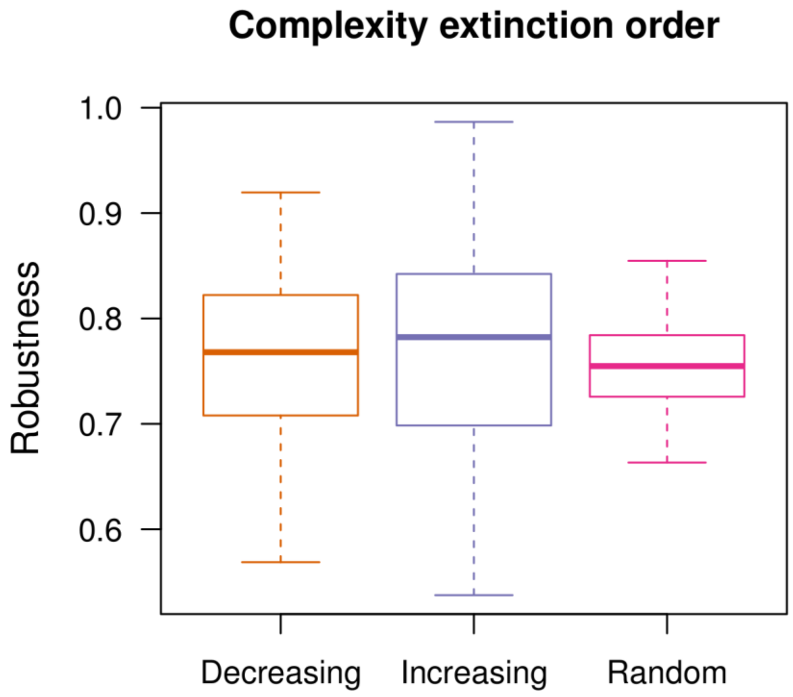

Supplementary Figure 3 | Robustness of digital networks against the removal of hosts in decreasing, increasing and random order of complexity. Host complexity was measured as the most complex of the host's tasks (see Methods). Boxes indicate first and third quartiles, whiskers indicate range values, and horizontal lines indicate median values.

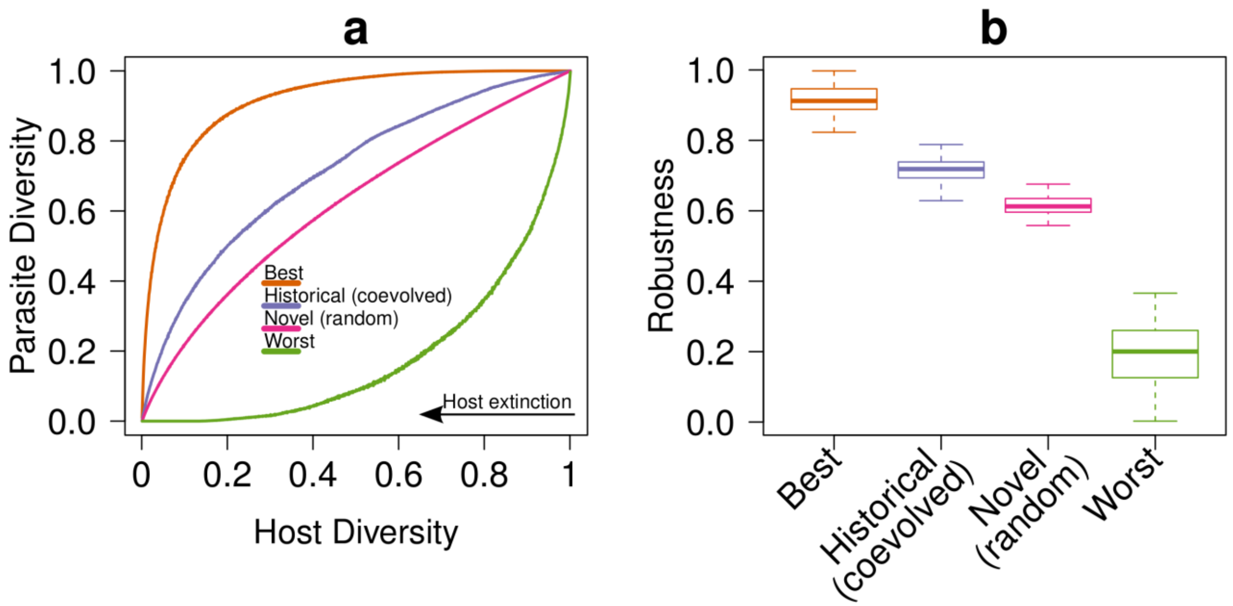

Supplementary Figure 4 | Results of host-removal experiments in digital networks obtained by using organisms' genotypes as taxonomic units (instead of tasks). **a**) Average fraction of parasite species remaining in the digital host-parasite network after the subsequent removal of all host species according to historical conditions (from right to left) in all the experiments, contrasted with a best and a worst-case scenario, and a novel (random) scenario. **b**) Robustness (the areas under curves in the four host removal scenarios) of the digital host-parasite networks under different scenarios of host removal. Boxes indicate first and third quartiles, whiskers indicate range values, and horizontal lines indicate median values.

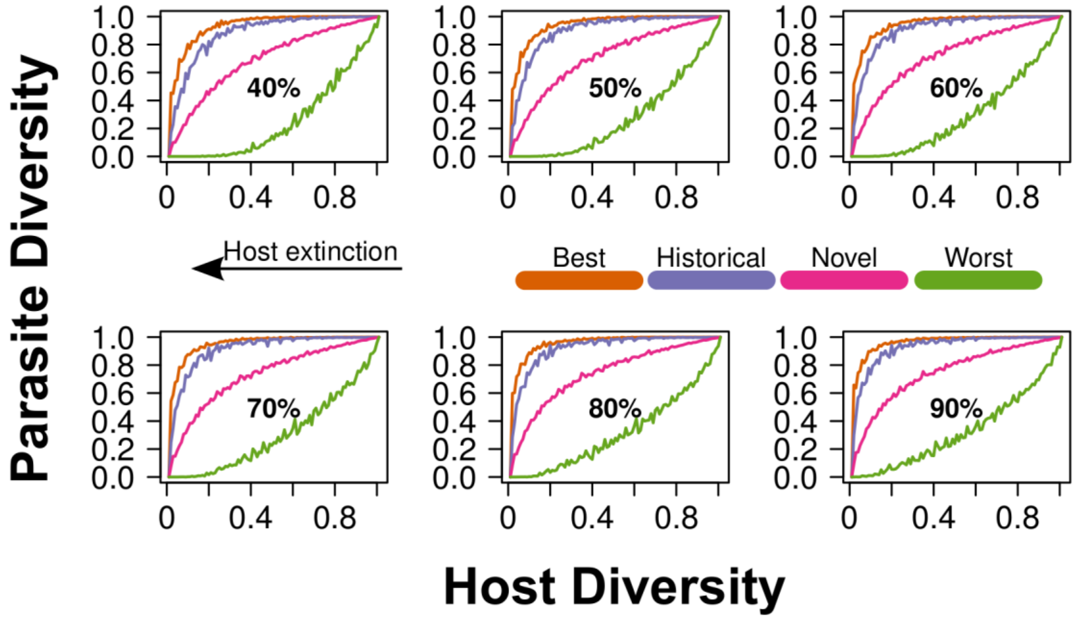

Supplementary Figure 5 | Disassembling partial networks. Average fraction of parasite species remaining in partial networks including random samples of 40%, 50%, 60%, 70%, 80% and 90% of the original edges after the subsequent removal of all host species according to the actual order of extinction observed in all experiments (historical), contrasted with a best-case and a worst-case scenario, and a novel (random) scenario.

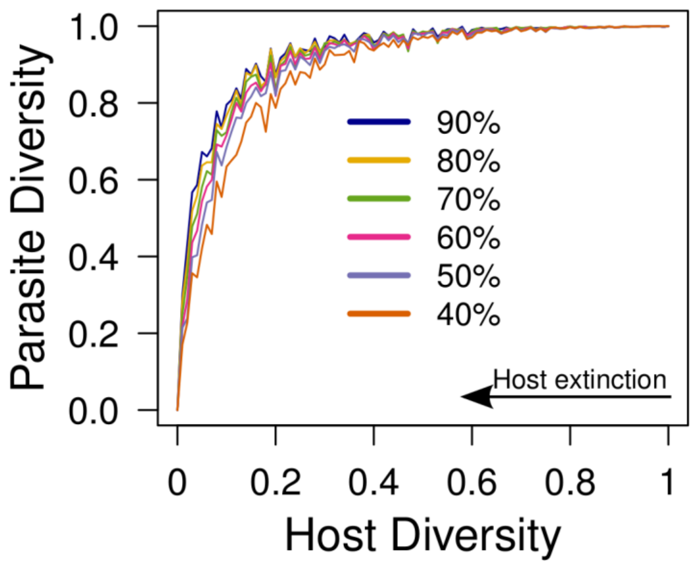

Supplementary Figure 6 | Comparison between historical disassembly in partial networks of increasing size. Average fraction of parasite species remaining in partial networks including random samples of 40%, 50%, 60%, 70%, 80% and 90% of the original edges after the subsequent removal of all host species according to the actual order of extinction (i.e., historical) observed in all experiments.

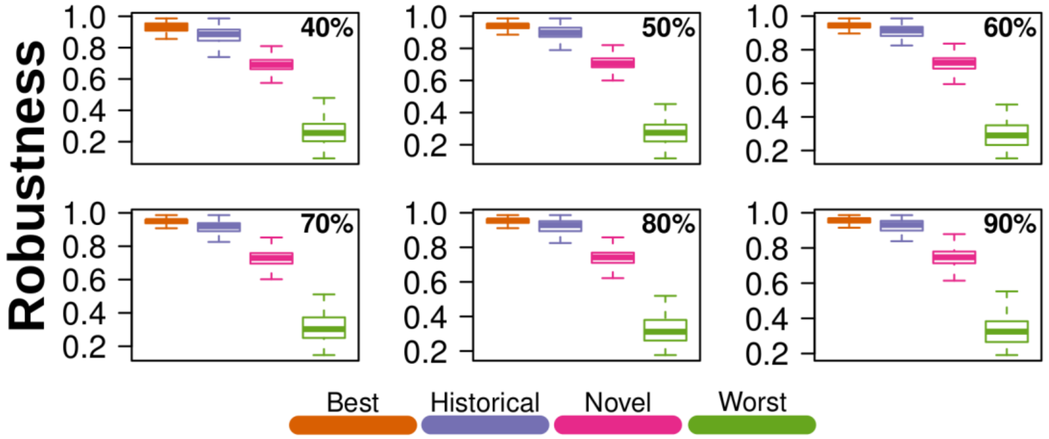

Supplementary Figure 7| Parasite assemblage robustness in partial networks. Boxplots of the robustness obtained in partial networks including random samples of 50%, 60%, 70%, 80% and 90% of the original edges resulting from the subsequent removal of all host species according to the actual order of extinction observed in all experiments, contrasted with a best and a worst case scenario, and a novel (random) scenario. Boxes indicate first and third quartiles, whiskers indicate range values, and horizontal lines indicate median values.

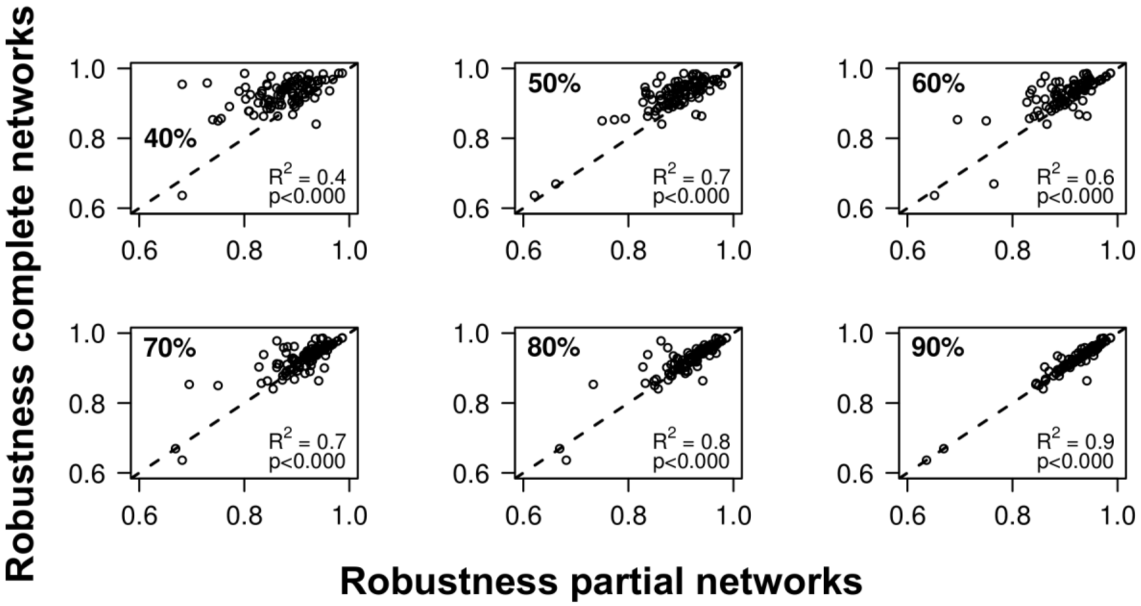

Supplementary Figure 8 | Comparison between parasite assemblage robustness in complete and partial networks. Relationships between the robustness resulting from disassembling the complete digital host-parasite networks according to the observed extinction risks and the corresponding robustness values obtained from partial networks including random samples of 40%, 50%, 60%, 70%, 80% and 90% of the original edges.

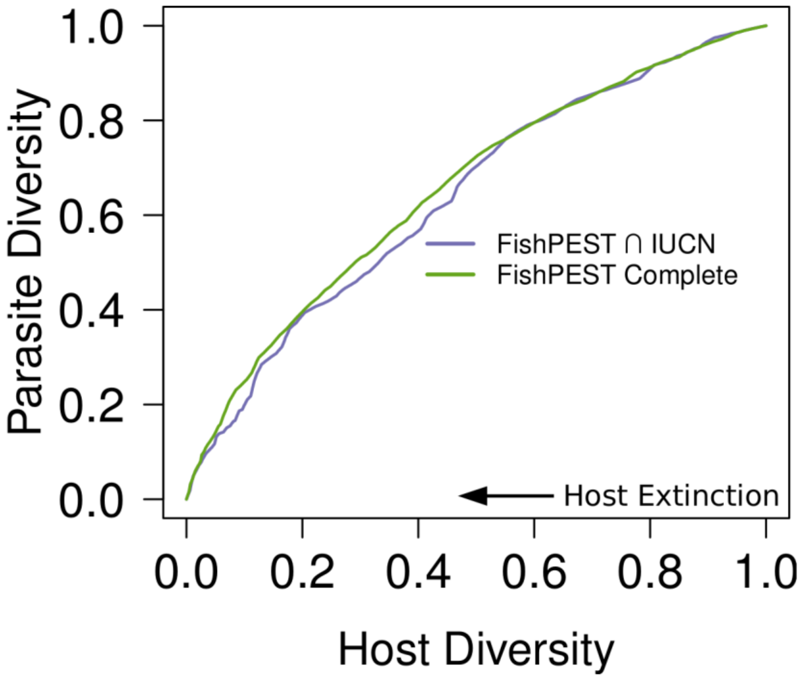

Supplementary Figure 9 | Effect of data availability on the estimation of global fish parasite assemblage robustness under historical conditions. Comparison between the historical robustness of the network based on the entire FishPEST database[13] and that of the same network filtered by excluding all host species not listed in IUCN red list, both disassembled according to intrinsic vulnerability values[15]. Disassembly was replicated 100 times randomizing the order of ties, and the results were averaged.

Supplementary Tables

Supplementary Table 1: Environmental parameters in the Avida simulations, and the resulting properties of communities and host-parasite networks at the end of the co-evolutionary phase. *Net resource availability* is the overall balance between in-flowing and out-flowing resource units per simulation step. *Carrying capacity* is the maximum possible number of simultaneously alive individuals, that is equal to the number of cells (which, in turn, is given by *World width* × *World height*). *Node Overlap/Segregation* and *Modularity* were computed according to the procedure described in Strona and Veech[36]. *Host/parasite interactions* corresponds to the number of links in the host/parasite networks.

	Min.	1st Qu.	Median	Mean	3rd Qu.	Max.	St. Dev.
Maturity (number of steps)	101000	175500	259500	264200	334250	491000	104609
Number of resources	1.00	4.00	5.00	4.74	6.00	8.00	1.78
Net resource availability	21.4	54.5	66.9	72.2	81.2	166.0	28.8
World width	50.0	65.0	83.0	83.8	104.0	119.0	22.0
World height	50.0	67.8	84.0	84.3	103.3	119.0	20.6
Carrying capacity	2862	5118	6671	7007	8270	13216	2390
Node Overlap/Segregation	-0.04	0.40	0.53	0.54	0.68	1.00	0.22
Modularity	0.00	0.59	0.66	0.62	0.74	0.85	0.20
Number of host phenotypes	1.0	15.0	21.0	23.1	27.3	65.0	11.6
Number of parasite phenotypes	2.0	7.8	14.0	21.6	31.0	80.0	19.3
Host/parasite interactions (phenotypes)	3	42	88	133	179	670	128
Number of host genotypes	843	1534	2025	2211	2730	5463	934
Number of parasite genotypes	58	398	712	872	1126	3472	640
Number of host individuals	2575	3961	5388	5666	6924	13018	2201
Number of parasite individuals	140	1317	2566	2790	3626	8968	1891

Supplementary Table 2: Relationships (expressed as Spearman's rank correlation coefficients) between the robustness of the digital host parasite networks obtained at the end of the co-evolutionary experiments, and the environmental features where the networks emerged (in terms of world dimensions and resource diversity/availability), network structural properties (node overlap/segregation, modularity, number of nodes, i.e. host and parasite phenotypes, and links, i.e. host/parasite interactions), genotypic and phenotypic diversity, and overall density of host and parasite individuals.

	**Robustness**			
	Historical	***Novel***	***Best***	***Worst***
Maturity (steps)	0.05	-0.06	0.04	0.05
Resources	-0.14	0.05	-0.12	0.02
Net resource availability	0.06	-0.08	-0.04	-0.05
World width	0.07	-0.05	0.20	-0.32
World height	-0.08	0.27	0.00	0.18
Carrying capacity	-0.07	0.14	0.08	-0.12
Node Overlap/Segregation **(raw value, Ń)**	0.25	-0.13	0.26	-0.12
Node Overlap/Segregation **(effect size,** Z_N**)**	0.13	-0.18	0.10	-0.10
Modularity	-0.27	-0.05	-0.31	0.01
Number of host phenotypes	0.06	0.16	0.16	0.11
Number of parasite phenotypes	-0.05	0.18	0.03	0.01
Host/parasite interactions (phenotypes)	-0.01	0.31	0.08	0.15
Number of host genotypes	0.01	-0.06	0.08	-0.36
Number of parasite genotypes	0.03	0.13	0.03	0.14
Number of host individuals	-0.01	-0.02	0.08	-0.32
Number of parasite individuals	0.05	-0.04	0.00	0.14
